# Venus Kinase Receptors at the Crossroads of Insulin Signaling: Their Role in Reproduction for Helminths and Insects

**DOI:** 10.3389/fendo.2015.00118

**Published:** 2015-08-03

**Authors:** Colette Dissous

**Affiliations:** ^1^Center for Infection and Immunity of Lille, INSERM U1019, University Lille Nord de France, Lille, France; ^2^CNRS-UMR 8204, Institut Pasteur de Lille, University Lille Nord de France, Lille, France

**Keywords:** venus kinase receptor, insulin, reproduction, helminths, insect vectors

## Abstract

Venus kinase receptors (VKRs) are invertebrate receptor tyrosine kinases (TKs) first discovered in the human parasite *Schistosoma*. They contain an extracellular Venus FlyTrap module similar to the ligand-binding domain of G protein-coupled receptors of class C and an intracellular TK domain similar to that of insulin receptors. VKRs are present from cnidarians to echinoderms. They were shown to be activated by amino-acids, to induce insulin-like intracellular pathways, and to be highly expressed in larvae and in gonads of helminths and insects. The function of VKR in gametogenesis was demonstrated in schistosomes by VKR silencing and recent studies in *Aedes aegypti* have confirmed the importance of VKR in mosquito egg formation. AaeVKR was shown to bind to ovary ecdysteroidogenic hormone and to activate the production of ecdysteroids by the ovary, independently of signaling mediated by insulin-like peptides. These new data confirm and specify the function of VKRs in the reproduction of helminths and insects and they open interesting perspectives for elucidating the role of VKRs in other models. VKR targeting would also provide opportunities for the control of parasites and various vector-borne infectious diseases.

## The Family of VKRs: A Brief History

The first venus kinase receptor (VKR) was discovered serendipitously in the platyhelminth *Schistosoma mansoni*. Working on the identification of insulin receptors (IRs) in this human trematode parasite, we isolated an IR-like transmembrane molecule with a tyrosine kinase (TK) domain similar to that of IR but with an unexpected Venus FlyTrap (VFT) module in its extracellular domain. This VFT module, usually found in many class C G protein-coupled receptors (GPCRs), was shown to be close to that of the GABA-receptor (1) and the new receptor was therefore named VKR (2). VKRs were subsequently discovered in other species and shown to belong to a new family of receptor tyrosine kinases (RTKs), present only in invertebrates from at least five phyla of the Bilateria branch (Platyhelminthes, Arthropoda, Annelida, Mollusca, Echinodermata) as well as to the Cnidaria phylum (2, 3). Such RTKs are found in a large majority of insects including several *Drosophila* species but exceptionally *vkr* genes are absent from the genome of *Drosophila melanogaster* as well as from the genomes of other species in the *melanogaster* subgroup. Not found in nematodes, *vkr* is also absent from the worm *Caenorhabditis elegans* (3). The absence of *vkr* genes in the two major invertebrate models, *D. melanogaster* and *C. elegans*, is probably the reason why the VKR family was not found earlier. A single *vkr* gene is usually contained in each invertebrate genome, except in trematodes and lepidopterans in which two copies of *vkr* have been identified (3, 4).

Phylogenetic studies have shown that within the RTK superfamily, the VKR family is close to that of IR (3). All VKR proteins exhibit highly conserved IR-like TK domains, suggesting that VKR and IR could transduce similar pathways. VFT domains of the various VKRs are less conserved in their putative ligand-binding site but their structure is similar to those of VFT-containing receptors which are able to bind small molecules. Accordingly, schistosome and insect recombinant VKRs were shown to bind amino-acids and their TK activity is induced preferentially by the extracellular binding of arginine at low (μM) concentrations (2, 4). As expected for RTKs in general, VKRs are active as dimers and they activate downstream components common to IR pathways, like PI3K/Akt/S6K and MAPKs (5–7).

Earlier investigations have indicated that VKRs are present in larval and adult stages of the parasite *S. mansoni*. VKRs were detected abundantly in germinal cells surrounding the neural mass of miracidia (the larvae released from embryonated eggs) and in female adults, *vkr* transcripts were found to be abundant in oocytes contained in the ovary and in the ovary duct (1, 5). Similarly, *vkr* transcripts were preferentially found in larvae or in female gonads of insects like *Tribolium castaneum, Apis mellifera, Anopheles gambiae* (2), and in the ovaries of *Aedes aegypti* (7). The presence of high levels of *vkr* transcripts in larval forms and in female gonads of these organisms already suggested the importance of VKR proteins in larval growth and differentiation as well as in reproduction (6).

## Insulin Signaling and VKR Function in Reproduction

Insulin and Insulin-like growth factor signaling (IIS) forms complex networks with other signaling pathways, especially with the amino-acid-sensing TOR (target of rapamycin)/S6K (p70 S6 kinase) pathway, to regulate nutrition, growth, development, longevity, as well as reproduction (8, 9).

In insects, evidence has been provided that IIS is involved in female reproduction in conjunction with juvenile hormone and ecdysteroid signaling pathways and that IIS controls vitellogenesis and oogenesis, thus coupling nutritional information to reproduction (10, 11). In *A. aegypti*, the primary vector of viruses infecting humans, the regulation of egg formation by IIS is well understood. Blood feeding triggers the release of two neurohormones from neurosecretory cells in the mosquito brain: ovary ecdysteroidogenic hormone (OEH) and insulin-like peptides (ILPs) (12, 13). OEH and ILPs stimulate the production by ovaries of ecdysteroid hormones that induce the secretion of yolk proteins by the fat body and their packaging into eggs (14–16). The gonadotropic role of ILP3 was clearly evidenced in the mosquito *A. aegypti*. Binding of ILP3 to IR expressed in mosquito ovaries stimulated the uptake of yolk proteins in oocytes and the production of ecdysteroid by ovaries confirming that the regulation of egg maturation was dependent on IIS in insects (11, 12).

In platyhelminths, more limited information has been obtained concerning the importance of IR receptors and IIS in development and reproductive activities. IRs have been characterized in a large panel of flatworms (17–19) and genome-wide searches of ILP in parasitic flatworms have recently reported for the first time the presence of two ILP genes (ILP-1 and ILP-2) in cestode genomes and one ILP gene in trematodes (20). Moreover, in the cestode *Taenia solium*, ILP-1 was shown to be predominantly expressed in ovarian tissues (20), a finding consistent with the hypothesis that insulin could play in flatworms as in insects a conserved role in the regulation of fertility and germ cell populations besides its role in metabolism.

In *Schistosoma japonicum*, immunization of mice with the insulin-binding domain of schistosome IR provokes a retardation of the growth of adult parasites and a substantial decrease of parasite egg maturation and laying in parasitized animals (21). Profound alterations in pairing and egg laying by schistosomes were also induced by drugs inhibiting the pathway of Akt, a major downstream kinase target of IR and a central player at the crossroads of signal transduction pathways activated in response to insulin (22). Additionally, the *in vitro* treatment of schistosomes with commercial IR kinase inhibitors, like tyrphostin AG1024 and HNMP-A3, led to dramatic effects on fertility and viability of larval and adult parasites (23). These data support a potential importance of IIS in reproduction of helminths, similar to its role in insects. However, since both AG1024 and HNMP-A3 drugs inhibit with a similar efficiency the kinase activities of schistosome IR and VKR (IR-like), which are both expressed in parasite ovaries, it was hypothesized that VKR was also a potential actor in helminth reproduction (23). The implication of schistosome VKRs in oogenesis and spermatogenesis was further demonstrated using RNA interference in adult worms. Results of SmVKR knockdown confirmed the importance of these receptors in germ cell differentiation and reproduction processes in helminths (5).

Recently, evidence has been given for the role of VKR in the reproduction of *A. aegypti* (7). AaeVKR is preferentially expressed in the ovaries of blood-fed adult females and its targeting by RNA interference in the mosquito disables egg formation when the latter is mediated by OEH. AaeVKR knockdown has no effect on ovary ecdysteroid production mediated by ILP3, and these data confirm a unique and specific role of VKR in the activation of egg formation in the mosquito (7).

Knowledge about the hormonal mechanisms that regulate schistosome reproduction still remains relatively sparse compared to insects. However, previous studies demonstrated that *S. mansoni* synthesize ecdysteroids (the insect ecdysone and 20 hydroxyecdysone) which are present in the juvenile worms, in the adults, and in the eggs (24). Moreover, an ortholog of the *Drosophila* ecdysone receptor SmE78 was identified in *S. mansoni* and shown to be highly expressed in the parasite eggs (25). Even though it has not yet been demonstrated that SmE78 could be involved in the transduction of an ecdysone signal required for the regulation of egg formation, it is tempting to hypothesize a putative function of schistosome VKR in the production of ecdysteroids similar to that shown in mosquitoes.

## New Insights into VKR Activating Ligands

Previous bioassays have shown that recombinant VKRs from the schistosome and diverse insects were able to autophosphorylate and to activate TK signaling upon the binding of amino-acids, and preferentially upon the binding of arginine. VFT modules constitute the binding pocket of various receptors activated by small molecules (26). They are composed of two lobes that close around the ligand and in most class C GPCRs, these modules contain the binding site for natural amino-acids or derivatives. In these receptors, ligand recognition is dependent on a consensus motif of eight residues that participates in the binding of the α-amino-acid group (27). The serine residue, which is essential for amino-acid binding in VFTs of class C GPCRs, is perfectly conserved in almost all VKRs and its presence was shown to be required for the activation of recombinant VKRs by arginine (3, 4).

However, VKRs exist (in cestodes for example) which do not possess the conserved serine residue (3) and which are not activated by amino-acids, suggesting that other molecules serve as ligands for VKR. Moreover, in *S. mansoni*, two VKRs exist and are activated independently by arginine or calcium ions (4).

The recent finding of Vogel et al. (7) has led us to reassess important issues about the deorphanization of VKRs and the deciphering of their mechanisms of activation. Indeed, interestingly, the authors demonstrate that the neurohormone OEH binds to the dimers of *A. aegypti* VKR expressed in S2 *Drosophila* cells and that OEH binding activates the recombinant AaeVKR and specifically stimulates Akt phosphorylation in the cells, indicating that OEH, a neurohormone required for egg formation in *A. aegypti* (13, 16), is a ligand for the mosquito VKR.

Ovary ecdysteroidogenic hormone belongs to a poorly characterized family of neuropeptides named neuroparsins. These molecules are known only in arthropods. They were isolated for the first time from the *pars intercerebralis-corpora cardiaca* complex of *Locusta migratoria* (28), and were then identified in a number of insects (29). Neuroparsins show a limited sequence similarity with vertebrate insulin growth factor binding proteins and it was suggested that they could act as ILP-binding proteins to modulate insulin signaling in arthropods. However, in *A. aegypti*, whilst the neuroparsin OEH effectively activates insulin signaling, this is independent of the IR (16) and occurs via its specific binding to another receptor that is very likely AaeVKR (7).

It seems therefore that VKR might be considered in arthropods as a “neuroparsin receptor”. The observation that members of the *D. melanogaster* subgroup lack both neuroparsin (30) and *vkr* (2) genes supports the conclusion that neuroparsin is the ligand for VKR in most if not all insects, but this has still to be confirmed. Of course, a big question remains open about the existence and the nature of unknown hormones which could potentially regulate the activity of VKR during the reproduction of other organisms, particularly of schistosomes. In these separate-sexed helminths, egg production by female worms requires a constant pairing-contact with a male. A male factor stimulating egg production has been extensively researched during the last decades but its nature still remains very elusive (31). Research for hormone or peptide ligands for VKR potentially contained in fluids secreted at the point of intimate contact between male and female worms should be attempted.

Additionally, the activation by OEH of AaeVKR introduces the concept that the VFT module of VKRs should be able to bind large molecules in addition to amino-acids or ions. Other examples of VFT-containing receptors, such as the ANF (Atrial Natriuretic Factor) guanylate cyclase-coupled receptor, are known to possess VFT modules that bind ions together with peptides or hormones that stabilize VFT dimerization and receptor activation (32). We suggest that a similar mode of allosteric regulation by specific protein molecules might be applicable to the various VKRs throughout the evolution.

**Figure 1:**
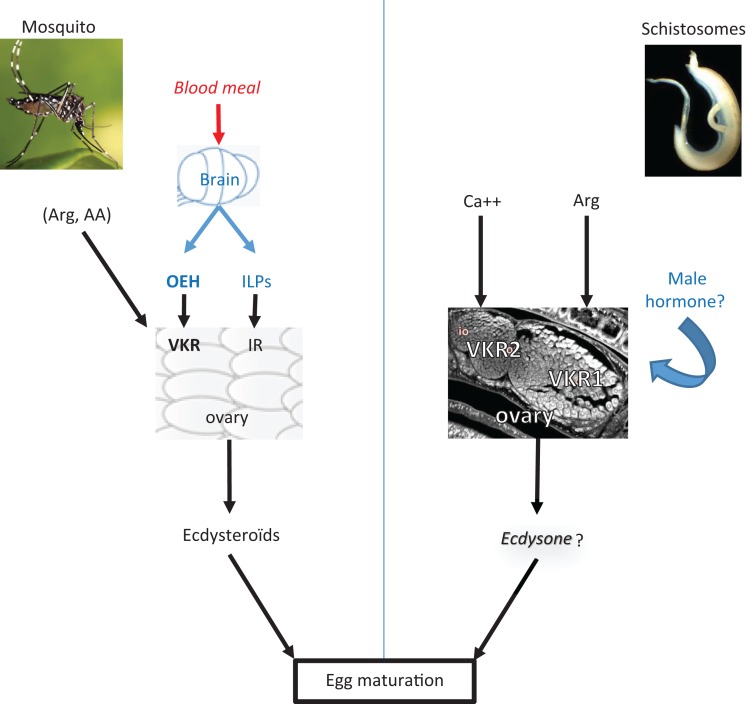
**VKR signaling in the reproduction of insects and helminths**. This scheme illustrates how VKR signaling participates in the activation of egg formation in mosquitoes as well as to egg maturation in helminths. In *A. aegypti*, blood feeding triggers the release from the mosquito brain of insulin-like peptides (ILPs) and ovary ecdysteroidogenic hormone (OEH) which bind, respectively, to IR and VKR in the ovary and activate the production of ecdysteroids necessary for egg formation (7, 12–14). Arginine and other amino-acids are also potential ligands able to activate the mosquito VKR (2). In schistosomes, VKR2 expressed in the immature part of the ovary is activated by calcium ions and VKR1 present in the big mature oocytes is activated by arginine, as well as very likely by a male hormone that still remains to be characterized. VKR1 is supposed to be involved in oocyte migration and egg assembly but the processes of egg formation and maturation are still unstudied in schistosomes (5).

In conclusion, recent information converges toward the importance of VKRs in the reproductive functions of helminths and insect vectors (Figure 1), providing therefore new perspectives for the control of human parasitic and infectious diseases. We can postulate that a strategy using antagonist ligands of the VFT domains to prevent their dimerization and VKR activation should find medicinal applications in the control of parasite and insect populations.

## Conflict of Interest Statement

The author declares that the research was conducted in the absence of any commercial or financial relationships that could be construed as a potential conflict of interest.
